# Supplementary material for: SNP Array Screening and Long Range PCR-Based Targeted Next Generation Sequencing for Autosomal Recessive Disease with Consanguinity: Insight from a Case of Xeroderma Pigmentosum Group C
Source: Genes (Basel). 2023 Nov 15;14(11):2079. doi: 10.3390/genes14112079 (PMC10671442; doi:10.3390/genes14112079)
Supplement: Supplementary file 1 [file genes-14-02079-s001.zip › genes-2690793-supplementary.pdf]

Supplemental Table S1. Primer sequences of long-range PCR and XPC mutation specific site.

Primer set	Primer Name	Primer sequence	Primer position (GRCh38.p14)	Product size (bp)
ERCC4-A Long	ERCC4_L_A-F	5'-TATCTGCCTCAGAGGAAACGCTGTATG-3'	13918667_13918693	18200
	ERCC4_L_A-R	5'-GAGCTGAATTAAGCACCTCCTCTGTCC-3'	13936866_13936840	
ERCC4-B Long	ERCC4_L_B-F	5'-CAGCTTGAAATTTACAGGGCGAGTAGG-3'	13935700_13935726	19318
	ERCC4_L_B-R	5'-CTCGATGGCGTGGACTGTTTGTTATAG-3'	13955017_13954991	
XPC-A Long	XPC_L_A-F	5'-ACCCTCAAGGAGCTCATTCTACTGTGG-3'	14179936_14179910	19155
	XPC_L_A-R	5'-GTTTCTTCTTGTACAGCATGGCACAGG-3'	14160782_14160808	
XPC-B Long	XPC_L_B-F	5'-CCAGCCTCTCAATTCTATGCTGTTGG-3'	14162464_14162439	18453
	XPC_L_B-R	5'-AGACCTGAGTTCTGAGCCAAAGATTGC-3'	14144012_14144038	
XPC Exon 2	XPC_Ex2-F	5'-GTTTGGAGACAGGTCATAGAGC-3'	14173165_14173144	412
	XPC_Ex2-R	5'-GATCCAATCTTCCATGGACCC-3'	14172756_14172776	

Supplemental Table S2. Detected variants in *XPC* and *ERCC4*.

Gene	Chromosome	POS (hg38)	dbSNP_ID	REF	ALT	HGVS_format*	GT	AD	DP	MAF
XPC	chr3	14145845	rs2229090	G	C	c.*96C>G	1/1	1,303	304	0.3733
		14145949	rs2228001	G	T	c.2815C>A p.(Gln939Lys)	1/1	0,397	397	0.5810
		14146199	rs2733532	T	C	c.2605-40A>G	1/1	0,306	306	0.5905
		14147647	rs2257984	A	G	c.2514+261T>C	1/1	2,289	291	0.5815
		14148737	rs2279017	T	G	c.2251-6A>C	1/1	0,138	138	0.5800
		14149147	rs542883216	A	G	c.2116-199T>C	1/1	0,246	246	0.0382
		14150149	rs76357945	G	A	c.2116-1201C>T	1/1	0,259	259	0.3324
		14151004	rs2607734	A	G	c.2115+1331T>C	1/1	0,257	257	0.5808
		14151433	rs2607736	A	G	c.2115+902T>C	1/1	0,248	248	0.5531
		14154514	rs34574722	G	GC	c.2033+1820dupG	1/1	0,284	284	0.5531
		14156487	rs2958057	A	T	c.1881T>A p.(Ala627=)	1/1	0,236	236	1.0000
		14158387	rs2228000	G	A	c.1496C>T p.(Ala499Val)	1/1	0,331	331	0.3322
		14163943	rs2061316	C	G	c.900+870G>C	1/1	0,184	184	1.0000
		14166208	rs1106087	C	A	c.622-623G>T	1/1	1,175	176	0.3302
		14169601	rs2733537	A	G	c.412+837T>C	1/1	0,141	141	0.3559
		14172947	NR	C	CA	c.218_219insT<br>p.(Lys73fsAsnfsTer9)	1/1	0,205	205	NR
ERCC4	chr16	13937663	rs31869	G	A	c.1812-103G>A	1/1	0,182	182	0.9825
		13945011	rs141025391	T	G	c.2017+176T>G	1/1	0,171	171	0.0224

* Reference sequences are NM_004628.5 for *XPC* and NM_005236.3 for *ERCC4*. AD; allele depth, ALT; variant bases, DP; depth, GT; genotype (1/1 means homozygous), MAF; minor allele frequency in Japanese women by Japanese multi omics reference panel (https://jmorp.megabank.tohoku.ac.jp/, last accessed 20 October 2023), NR; not reported, REF; reference base.
